# Two-layer modular analysis of gene and protein networks in breast cancer

**DOI:** 10.1186/1752-0509-8-81

**Published:** 2014-07-05

**Authors:** Alok Srivastava, Suraj Kumar, Ramakrishna Ramaswamy

**Affiliations:** 1 C R RAO Advanced Institute of Mathematics, Statistics and Computer Science, University of Hyderabad Campus, Hyderabad 500046, India; 2 Indian Institute of Technology Bhubaneswar, Bhuwaneswar 751013, India; 3 University of Hyderabad, Hyderabad 500046, India; 4 School of Integrative and Computational Sciences, Jawaharlal Nehru University, New Delhi 110 067, India

**Keywords:** Protein-protein interaction, Gene expression, Networks, Modules, Gene ontology

## Abstract

**Background:**

Genomic, proteomic and high-throughput gene expression data, when integrated, can be used to map the interaction networks between genes and proteins. Different approaches have been used to analyze these networks, especially in cancer, where mutations in biologically related genes that encode mutually interacting proteins are believed to be involved. This system of integrated networks as a whole exhibits emergent biological properties that are not obvious at the individual network level. We analyze the system in terms of modules, namely a set of densely interconnected nodes that can be further divided into submodules that are expected to participate in multiple biological activities in coordinated manner.

**Results:**

In the present work we construct two layers of the breast cancer network: the gene layer, where the correlation network of breast cancer genes is analyzed to identify gene modules, and the protein layer, where each gene module is extended to map out the network of expressed proteins and their interactions in order to identify submodules. Each module and its associated submodules are analyzed to test the robustness of their topological distribution. The constituent biological phenomena are explored through the use of the Gene Ontology. We thus construct a “network of networks”, and demonstrate that both the gene and protein interaction networks are modular in nature. By focusing on the ontological classification, we are able to determine the entire GO profiles that are distributed at different levels of hierarchy. Within each submodule most of the proteins are biologically correlated, and participate in groups of distinct biological activities.

**Conclusions:**

The present approach is an effective method for discovering coherent gene modules and protein submodules. We show that this also provides a means of determining biological pathways (both novel and as well those that have been reported previously) that are related, in the present instance, to breast cancer. Similar strategies are likely to be useful in the analysis of other diseases as well.

## Background

The current paradigm in a *systems* approach to biological phenomena is that of networks and the interactions among them. Advances in genomic, proteomic and high-throughput gene expression data, when integrated, can be used to map the interaction networks between genes and proteins, as well as their association with specific biological activities. It has also become increasingly clear that an integrated analysis of these extensive components is crucial, especially in the case of cancers [1]. Mutation in biologically correlated genes affects the translation of key proteins that do not function in isolation: distinct biological activities are the result of the coordinated action of multiple proteins [2] and a reduction in the synthesis of one protein can directly affect various specialized biological actions.

In the case of breast cancer for example, the interaction network of 6004 proteins is, in different combinations, associated with 5732 biological processes (BP), 1930 molecular functions (MF) and 879 cellular components (CC) as specified in the Gene Ontology Annotation (GOA) [3] database. On this scale it is difficult to interpret the organization principle of such networks that may be composed of thousands of structural subunits. The more highly connected subunits participate in multiple biological activities [4].

The alternative *bottom-up* approach, namely breaking up the complex network into several interacting sub-networks, can be more helpful. These sub-networks help to analyse the activity at various levels of specificity, especially in case of complex diseases where the main interest is to elucidate the coordination principle that controls the progression of the disease.

The modular nature of a wide variety of complex networks has been investigated in detail in recent years, ranging from social networks [5], cellular phone networks [6], collaboration networks [7], citation networks [8], gene co-occurrence network [9], protein-protein interaction (PPI) networks [10], and metabolic networks [10]. A module can be defined as a subset of the nodes such that nodes within the module are densely connected while being sparsely connected with nodes in other modules [11]. Modules are the building blocks of higher-level functional organization, and can exhibit hierarchical properties. In particular, modules can be recursively divided into smaller submodules; such submodules are potentially a rich source of information on biological networks [12-15]. Nodes within a submodule are more likely to have closely related biological properties, and thus separating a network into modules and submodules can make it possible to understand the more specific domain of activities in which they participate, either singly or in a coordinated manner. (By domain we mean here the specializations defined by Gene Ontology (GO) [16], namely BP, MF or CC.)

**Figure 1:**
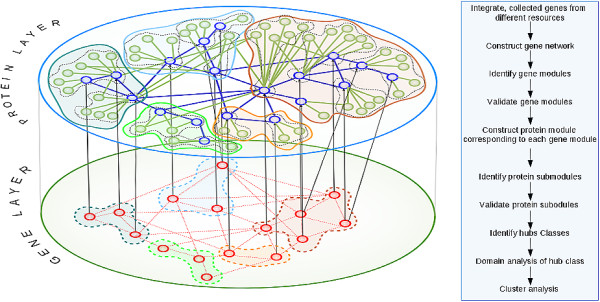
**Schematic diagram representing two-layer network structure between genes and proteins.** Nodes are coloured red in the gene layer. Each dotted hull shows an individual gene module of connected genes, based on their co-expression correlations. This is projected onto the protein layer to construct a primary network of expressed proteins, shown in blue. The secondary network of interacting proteins is highlighted in green. Each solid hull represents the protein module corresponding to the gene module. Protein submodules are shown within these modules. The accompanying flow-chart gives an overview of the protocol.

In the present paper we propose a general framework for the analysis of breast cancer data. By combining information on correlated genes and knowledge of their associated proteins, it is possible to construct a model two-layer network that can be explored to uncover the underlying biological phenomena in cancers. Analyzing the network as a whole gives insight into all annotated biological activities and specific sub-activities within the cell. On the *gene layer* we construct a correlation network from the expression data available for disease specific genes using Graphical Gaussian Modeling (GGM) [17]. The corresponding protein layer is constructed from the known set of proteins expressed by each of the genes to make the primary network, and a secondary network that shows their corresponding interactions is constructed from available protein interaction data. This two layer analysis is schematically shown in Figure 1. On each layer, modules are identified through a fast greedy optimization algorithm [18] applied recursively. We also investigate the robustness of the various modules by examining the effect of node removal. Each protein submodule is further divided into an inter-modular bridge class and an intra-modular group class, so as to identify the domains with the largest number of participating proteins. The overall aim of the present procedure is in effect to construct a network of networks and to examine the connections between the different domain groups within the protein submodules.

There are several methods that can be used to detect modularity, and a number of algorithms have been described [10,18-24]. In our study we focus on the gene module and protein submodule detection using a fast greedy modularity optimization technique [18] that is efficient in the analysis of large networks. The detailed topology of the submodules is interesting: the group of proteins within a submodule that interact physically with other proteins tend to form two sorts of hubs. The so called ‘party hubs’ interact with most (or all) of their partners simultaneously, while ‘date hubs’ are those that interact with different partners at different times [25]. Party hubs are intra-modular and the constituent proteins are believed to perform a specific biological activity within the submodule. In contrast, the activity of date hubs is intermodular and link different biological domains [26]. Proteins belonging to each hub within a submodule can be further explored from an ontological perspective: a group of proteins can be located in one or more cellular component, be active in one or more biological processes, and perform one or more molecular functions, but since domain activity is shared by several (and overlapping) groups, classification using GO terms can become very complex. This can be reduced to some extent by the implementation of a majority rule or by GO homogeneity (GO-H) analysis [27]. In the present case we use this latter reduction to identify GO terms associated with the largest fraction of proteins within a given hub class (i.e. party or date).

A domain cluster is a group of three or more GO terms connected together by a *parent–child* relationship, namely these form a directed acyclic graph (DAG) [16]. GO terms up to the 2nd level is general while terms at the lower levels are specific, and we explored the biological domain cluster of similar GO terms through OntoVisT [28], a general purpose tool developed earlier for interactive visualization and navigation of any ontology.

We present our findings using a exhaustive list of breast cancer genes, proteins and their PPI network from the Human Protein Reference Database (HPRD) [29] in combination with the comprehensive and well-established microarray datasets from breast cancer patients [30,31].

## Methods

### Materials: breast cancer data

We have integrated nine cancer resources in order to obtain a comprehensive list of breast cancer marker genes. The different resources focus on different aspects of biology:

• NCG (Network of Cancer Genes) has data on gene mutations [32],

• TGDBs (Tumor Gene Family of Databases) lists the target genes implicated in cancer-causing mutations [33],

• CGW (Cancer Genetics Web) details primary mutations that cause cancer, as well as secondary genetic abnormalities caused by cancer [34],

• CGC (Cancer Gene Census) catalogues all genes whose mutations have been implicated in cancer [35],

• KEGG (Kyoto Encyclopedia of Genes and Genomes) integrates current knowledge on molecular interaction networks [36],

• BCGD (Breast Cancer Gene Database) collects molecular genetic data related to genes involved in breast cancer [37],

• CGAP (Cancer Genome Anatomy Project) lists gene expression profiles of cancer cells [38],

• OMIM (Online Mendelian Inheritance in Man) is a compendium of information on genetic disorders and genes [39], and

• GAD (Genetic Association Database) contains genetic association study data reported in the literature [40].

From the above integrated sources we identified 975 breast cancer genes of which 956 are unique (since some genes have more than one identifier [41]). This is a more exhaustive list than what has been hitherto available, and is summarized in Table 1. Details of the complete marker gene list are available in Additional file 1: Table S1.

**Table 1:** Breast cancer gene information

**Data source**	**BC genes**	**Processed genes**	**Version**	**Reference**
KEGG	9	9	Jan, 2010 (V-53)	WGS
CGC	19	20	Apr, 2011	LC
BCGD	62	62	1999	LC
TGDB	67	67	1999	LC
CGAP	69	69	Jan, 2010	GE
CGW	82	83	ApR, 2003	LC
NCG	140	144	Jun, 2011 (V-2.1)	HTMS
GAD	695	690	Jun, 2011	LC
Total	975	956		

WGS, Whole genome sequencing; LC, Literature curation; GE, Gene Expression; HTMS, High throughput mutational screening; V, Version;

### Gene expression microarray data

We use the comprehensive and well curated microarray data sets first studied by West et al. [30] and Gyorffy et al. [31], using UniProt [41] in order to convert synonymous gene symbols to their current approved labels. These datasets are termed Set 1 and Set 2 respectively throughout this paper. Samples with more than 20% missing values were discarded, and the standard *K* nearest neighbor (KNN) imputation technique [42] with *k =* 10 was used to estimate the missing values in the other samples. Imputed data was then averaged over all replicates to obtain a processed data matrix; the first dataset thus contains 49 breast tumor samples separated into two classes: 25 positive samples for estrogen receptor (ER+) and 24 negative (ER-) with expression levels measured for 5728 genes. The second dataset has 1809 breast tumor samples separated into three classes: 295 HER2+ samples, 1285 ER+/HER2- samples, and 229 ER-/HER2- samples with expression levels measured for 12496 genes. The normalized expression for the ESR1 gene (Affymetrix ID 205225) at above 500 is considered as ER+, while ERBB2 gene (Affymetrix ID 216836_s) above 4800 is regarded as HER2+ [31].

### Gene-protein association and protein-protein interaction data

In order to construct the primary network of expressed proteins we map genes to the UniProt (July 2011) dataset [41] that lists 41149 proteins expressed by 19511 genes. The primary network is further extended to form a secondary network of interacting proteins using HPRD (Release 9, Aug 2011) [29]. This database has two classes of interaction data: binary (if two proteins interact directly) and complex (when several proteins form a complex). Since protein complexes also constitute functional groups and take part in activation or inhibition [43] we also included these complexes in our study with the assumption that all proteins in a complex interact with each other. There are a total of 91029 binary interactions between 13494 proteins and 1521 protein complexes that are associated with 3652 proteins. On combining both, we obtain 13691 proteins, with a total of 110613 interactions.

### Gene ontology annotation data

GO data was used to explore the biological activity of a group of proteins, by analyzing the molecular functions (MFs) which they perform, the biological processes (BPs) in which they participate and their cellular components. The ontology consists of over thirty thousand terms distributed across 12 different levels of hierarchy, starting from generic terms at the highest level, to more specific terms at the leaf nodes. To incorporate the biological domain knowledge to the entire groups of proteins, we used GOA data (July 2011) [3] and found that the 40422 proteins are mapped to 7488 GO:BP terms, 3221 GO:MF terms, and 1053 GO:CC terms respectively.

### Methods

#### Gene network using graphical Gaussian modeling

Microarray data for the integrated set of breast cancer genes from different resources were used to construct the gene-gene interaction network based on graphical Gaussian modeling (GGM) [17]. The false discovery rate (FDR) criterion is then chosen to filter out the least significant genes, thus providing a computational criterion that permits us to determine which edge is to be included in the network. To construct the gene network based on the GGM algorithm, we used the Gene Net algorithm that is included in the R package [44].

### Modularity optimization using fast greedy technique

The gene network is further analyzed to identify modules using the CNM fast greedy optimization technique [18]; this identifies a subset of nodes that are densely connected within modules while being sparsely connected to nodes in other modules. We use the igraph implementation contained in the R package [45] to identify gene modules and protein submodules.

### Leave-one-out cross-validation (LOO-CV) and majority-voting technique

To test the robustness of the modular structure obtained above, we implemented the standard (but computationally expensive) LOO-CV technique by removing a node and its corresponding interactions from the data to predict the changed module distribution. The process is repeated iteratively for each node in the complete network, and majority voting is then used to identify the most robust modules.

### Protein network construction

Each gene module is projected onto the protein layer in order to construct the primary network of expressed proteins and further extended to include the secondary network of interacting proteins. Combining both primary and secondary network components gives the two-layer network shown in Figure 1. Each protein module is then further analyzed to identify the submodules, using the CNM algorithm along with LOO cross validation.

Each protein submodule obtained above is divided into bridge (B) and group (G) hub classes, and further analyzed using GO homogeneity (GO-H) to identify the domain groups of those proteins that have a majority participation in specific GO categories.

### GO analysis

For a group of proteins contained in a hub class of submodule *i*, GO Homogeneity (GO-H), is defined as a GO term that has the maximum fraction of proteins, among all the mapped GO terms.

**Figure 2:**
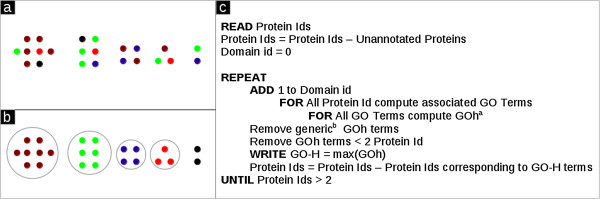
**Domain analysis for each hub class of protein submodule. (a)** Schematic of the group of proteins, **(b)** the GO domain representation of several groups of proteins, and **(c)** the Pseudocode for assigning GO description to a given protein list;. a defined as the fraction of protein contained in a GO term, b defines term < 2nd level and GO-H represent the max fraction of protein represented by a GO group.

GO-H_i_ = max_j_ [n^j^_i_/n_i_] = max_j_ [GO h^j^_i_ ]

where *n*_*i*_ is the number of proteins in the group *i* that have any GO annotations, and *n*^*j*^_*i*_ the number of proteins that have a specific GO term *j. GOh*^*j*^_*i*_ represents fraction of protein contained in group *j*. After assigning *n*^*j*^_*i*_ proteins to a GO term *j*, *n*_*i*_*- n*^*j*^_*i*_ proteins still remain and these may participate in other groups. This is done iteratively for the complete list as shown in Figure 2 so as to assign GO terms as well as group labels to all the remaining proteins.

Unannotated proteins introduce errors when included in GO-H analysis, and are therefore removed from the list. In order to identify the domain cluster represented by a group of GO terms we use OntoVisT [28] which as mentioned above, is a general purpose ontological visualization tool.

## Results

As indicated in the Materials section we combined 9 different cancer resources to get 956 breast cancer marker genes, of which 610 and 840 genes were found to be present in Sets 1 and 2 respectively. These are then used to construct the gene layer networks for detailed analysis.

### Gene layer construction and analysis of gene modules

We construct a partial correlation based gene network using the GGM technique. For each edge the partial correlation (pcor), p-value (pval), FDR (qval) and corresponding posterior probabilities (prob) are calculated, and the FDR criterion is employed to assess its significance. Table 2 summarizes the information regarding the gene layer network. The sparse nature of these networks with a fairly low number of genes results in a low modularity score *Q*_*max*_*,* for the optimal partitions.

**Table 2:** Summary of gene layer network for both datasets of breast cancer genes

	**Set 1: West et al. **[30]			**Set 2: Gyorffy et al. **[31]			
	**All samples**	**ER+**	**ER-**	**ALL samples**	**HER2+**	**ER+/HER2**	**ER-/HER2-**
Number of samples	49	25	24	1809	295	1285	229
Number of genes	610	610	610	796	796	796	796
λ	0.80	0.81	0.83	0.03	0.14	0.04	0.18
qval	0.30	0.30	0.30	0.30	0.30	0.30	0.30
Genes	513	464	459	796	791	789	793
Number of interactions	4619	5718	1960	3488	6086	2745	7546
α	1.23	1.37	1.38	1.35	1.25	1.40	1.21
Qmax	0.25	0.19	0.47	0.35	0.23	0.39	0.23
Number of modules	7	7	10	10	7	11	6
% accuracy, LOO-CV	78	71	60	57	61	76	72
Similarity measure		86	74		87	83	88

λ, optimal shrinkage intensity; qval, cut off for the partial correlation (the threshold is adjusted so as to ensure that all well-studied breast cancer marker genes are included in the networks); α, power-law exponent; Qmax, maximum modularity score; LOO-CV, Leave-one-out cross-validation.

For Set 1 a total of 4619 edges are found with a significance level of 30% FDR among the 513 genes (complete data given in Additional file 2: Table S2). The data was further stratified into ER + and ER- subtypes, and the same analysis was carried out (see Additional file 2: Tables S2). For Set 2, there were a total of 3488 edges at the same significance among the 796 genes (see Additional file 3: Table S3), and the samples in Set 2 were stratified into the three subtypes HER2+, ER+/HER2- and ER-HER2- as discussed in the Materials and Methods section. The analysis for the stratified samples gives the result presented in Table 2.

The networks and modules that result from the present analysis are consistent across stratification with some variation. Consider Set 1. When all the samples are taken together, we find 7 modules with a modularity score of 0.25. Most of the genes are included in both the ER + and ER- sample networks, and the results for the subtypes share a high similarity (86% and 74%, respectively) with the results for the total sample set. The number of modules for ER- is slightly larger and may indicate an increased heterogeneity of the subtype, but the modules themselves are quite similar.

In Set 2, stratification again gives results that are fairly consistent, with the number of genes being nearly constant across the subtypes. The number of modules varies although the similarity measure again gives a high level of concordance between the modules obtained by considering the entire sample set and the subtypes (87%, 83% and 88% respectively).

Cross validation results (LOO-CV followed by majority voting) indicate that overall, 78% of the modular structure remains intact for Set 1 (71% for the ER + subtype and 60% for ER-) while only 57% is preserved for Set 2 (and correspondingly 61% for HER2+, 76% for ER+/HER2-, and 72% for ER-/HER2-). See Table 2. Some mismatch might be expected due to the removal of hubs or some other important nodes from the network, but on the whole, these results reliably show that the breast cancer gene network is modular in nature.

### Protein layer construction and analysis of protein submodules

Modules that are identified in the gene layer for entire sample set are mapped to their expressed proteins. This gives the primary protein network, and the secondary network is then constructed by examining all proteins that interact with the primary network. Both networks when combined give the protein module that corresponds to each gene module. Together this forms the protein layer, as explained in Figure 1. We remove loops and multiple edges prior to application of the CNM algorithm to obtain submodules in the protein layer.

Our results for Set 1 are summarized in Table 3. There are 7 protein modules, each corresponding to a gene module and these are numbered by rank. The degree distribution of the networks in each protein module follows a power-law, with exponents between 1.5 and 1.8. The protein modules are much larger than the corresponding gene modules and these also have a high interaction density (namely the number of interactions per module). Q_max._ varies from 0.632 for module 3 which has a large number of proteins and correspondingly the most interactions, to 0.798 for module 6, a sparse graph of relatively fewer proteins. Q_max_ is inversely correlated to the number of protein-protein interactions. Module 7 has only 7 genes associated with a sparse graph of 113 interactions among 114 proteins. Redoing our analysis using LOO-CV suggests that more than 86% of the sub-modular structure is robust. The smallest modules, 6 and 7 give the same submodule structure regardless of which nodes are left out. These modules are very sparse and do not contain any hubs. In the larger modules where there are hubs, removal of such a node can give very different submodules although the average Q_max_ value does not change significantly, indicating that modularity is a robust property.

**Table 3:** Summary of topological and biological analysis of gene module and their corresponding protein modules for Set 1

**Module Id**	**1**	**2**	**3**	**4**	**5**	**6**	**7**	**#U Sum**
**Topological analysis**								
**Genes**	142	142	125	40	35	22	7	513
**Expressed proteins**	640	486	439	159	152	79	10	1958
**PPI**	7465	6576	8163	2106	1317	885	126	24752
**Processed protein in PPI**	3284	3206	3187	1338	797	575	114	6519
**Processed PPI**	6951	6095	7712	1985	1250	794	113	23173
**α**	1.606	1.632	1.576	1.701	1.658	1.698	1.777	
**Qmax**	0.637	0.662	0.632	0.714	0.792	0.798	0.793	
**Modules (Isolated modules)**	309 (7)	259 (4)	28 (11)	26 (10)	23 (8)	17 (7)	6 (4)	155 (51)
**%age accuracy, LOO-CV**	86.510	87.410	95.199	99.028	99.624	100	100	
**Avg. Qmax, LOO-CV**	0.639	0.664	0.634	0.714	0.797	0.797	0.791	
**Module range, LOO-CV**	24-33	20-28	24-34	25-32	21-25	16-18	5-6	
**Avg. Modules, LOO-CV**	28	26	28	26	22	16	5	
**Biological analysis**								
**GO:BP, Groups**	137	135	111	71	45	34	6	539
**GO:BP, Terms**	96	117	96	67	47	37	8	205
**GO:BP, Referenced**	42	54	46	32	25	19	8	85
**GO:MF, Groups**	131	124	115	69	44	35	7	525
**GO:MF, Terms**	79	72	72	51	33	30	10	145
**GO:MF, Referenced**	55	40	43	34	24	23	8	91
**GO:CC, Groups**	112	95	92	55	42	32	6	434
**GO:CC, Terms**	15	14	14	11	10	13	6	23
**GO:CC, Referenced**	12	11	12	9	8	9	6	16

Topological analysis represents the analysis of network topology, while biological analysis represents the domain analysis of individual modules; Genes, Number of genes; Expressed Proteins, Proteins expressed by genes in module; PPI, Number of interaction between proteins; Processed protein in PPI, Number of proteins after removing loops and multiple edges; Processed PPI, Number of interaction in processed PPI; α, Power law exponent; Qmax, maximum modularity score; LOO-CV, Leave-one-out cross-validation; Avg. modules, Mean number of modules in LOO-CV; GO:BP, GO:MF, GO:CC represent the GO biological process, GO molecular function, GO cellular component respectively, while Groups represents the domain groups annotated with each module, Terms represent the unique number of GO term; and Referenced represents GO terms previously reported in literature, #U Sum, Unique sum.

Set 2 when similarly analysed (see Additional file 4: Table S4) gives 10 protein modules with similar power-law distribution in degree, with exponent in the same range, 1.58-1.75. The Q_max_ value shows larger variation, from 0.62 to 0.87, and the number of submodules vary from 10 to 40 with considerable inter-module linkage. In other aspects such as modularity robustness, this dataset is similar to Set 1.

### The biological properties of submodules

Proteins in each submodule can be divided into two classes: group proteins (G) that form so—called party hubs that interact primarily with other proteins within the same submodule, and bridge proteins (B) which are the “date” hubs that interact with proteins in two or more submodules. Their associated domain properties are obtained by first mapping them to the GOA [3] followed by GO-H analysis. Generic GO terms are discarded up to the second level in the hierarchy, as also terms with fewer than three proteins. Each domain group of a submodule class is identified by a group identifier (ID) which is a set of three integers written *a.b.c,* with *a* representing the module ID, *b* the submodule ID and *c* the domain ID assigned by our algorithm.The pseudocode is given above (see Figure 2). Each group ID is also associated with a group label G, B, OG or OB representing group, bridge, overlapping groups or overlapping bridges, based on their topological class category. To identify clusters represented by a given set of GO terms we use OntoVisT [28]; PUBMED is also queried in order to determine whether the GO term in the submodule has been previously reported as occurring in any breast cancer related study.

Both datasets are studied in order to infer the biological activity for different hub classes of each submodule. The three GO categories BP, MF, and CC are separately analyzed. The detailed analysis of the main cluster of each GO category in Set 1 is presented here, and results for the other clusters of both sets are provided in the supplementary information.

### Analysis of molecular functions

**Figure 3:**
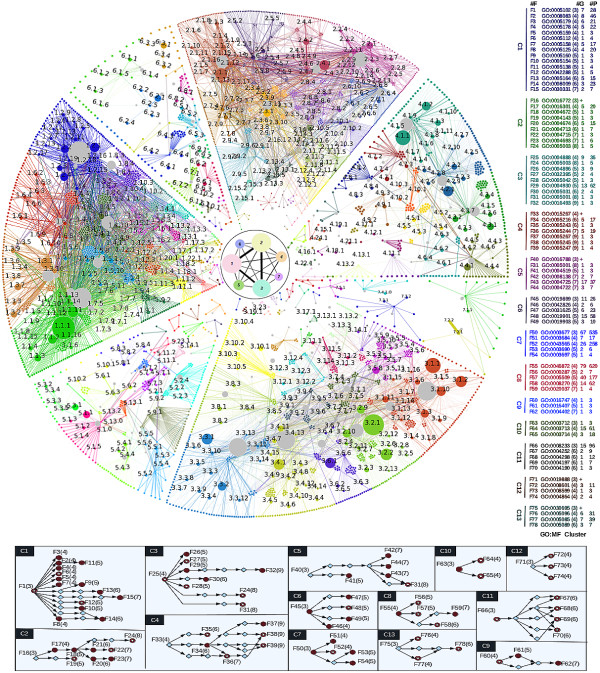
**Two layer network analysis of GO:MF for West dataset.** Inner circle shows the coarse grained gene modules while each of the outer sector represent the protein module corresponding to each gene module. Each submodule in the protein module is highlighted by different color and three digit number indicates the functional class description of each group of proteins. Functional clusters are shown in the right, contains function id (#Id), GO term followed by the depth in the GO hierarchy in brackets, number of groups (#G), and also number of proteins (#P) contained in each specific GO:MF term. First term in each cluster represents the cluster description. Detailed description of each cluster is discussed in the bottom panel. Disc, ring, and rhombus represents, GO:MF terms have already been reported in literature, newly reported GO terms and cluster component not reported in our analysis (shown with symbol ‘+’), respectively.

The two layer network deduced from the functional analysis of breast cancer genes found in Set 1 is shown in Figure 3. The inner circle depicts a coarse-grained image of the 7 modules in the gene layer. Details of the gene module and the protein submodules have been discussed above, and these are summarized in Table 3. The outer circle represents the protein layer, each sector representing the corresponding protein modules. Using HPRD [29] we have identified 23173 distinct protein-protein (PP) interactions in the network of 6519 proteins that are divided into 7 modules and 155 distinct submodules of which 51 are isolated. The interaction network in each sector shows the submodular network of the functional groups; these are highlighted in different colors. Each submodule is divided into group and bridge categories, based on their hub property, and these are then further analyzed for their functional associations using GO-H scores. Generic GO terms up to the second level in the hierarchy are discarded, as also those terms corresponding to fewer than three proteins. This results in 525 different functional groups that participate in 145 GO:MF terms (see Table 3). Each functional group category is marked with three digit identifier as discussed, and the details are included in Additional file 5: Table S5.As can be seen in Figure 3, namely the cluster diagram, there are 73 GO:MF terms from 13 different functional clusters. Among these, the term “Receptor binding” gives the largest cluster. There are 15 terms in panel C1 and several of these have been reported in earlier breast cancer studies. For illustrative purposes, some of these are discussed briefly below.

For instance, thioredoxin, a redox protein with growth factor activity (F2^++^) increases cell proliferation of breast cancer cells [46]. Many cell line models have been used to identify genetic elements that mediate the progression of breast cancer, both hormone dependent as well as hormone independent metastatic growth [47]. Integrin binding (F4) plays a crucial role in breast cancer tumor growth and metastasis [48]. EGFR binding (F10) is mediated through the binding of a mitogenic peptide epidermal growth factor (EGF) to a surface membrane receptor, EGFR of breast cancer cells [49]. Down-regulation of Interleukin-6 and its receptor (F11) results in growth inhibition of MCF-7 breast cancer cells [50], while high affinity of Insulin receptor binding (F7) has been observed in the same cells [51]. TGFβR binding (F9) regulates insulin-like growth factor binding protein (F5) (IGFBP)-3 production, which is a major antiproliferative factor and a key element for TGFβ-induced growth inhibition in breast cancer cells [52]. The breast cancer suppressor gene tyrosine kinase (PTKs) (F13) is involved in TNF-activated receptor activity by interacting with ‘TNF receptor-associated factor interacting protein’ (TRIP) in breast epithelial cells [53]. ER binding (F15) profiles are used to predict breast cancer outcome [54]. In some cases, breast cancer survivals shows elevation in the serum marker associated with proinflammatory cytokine activity (F8) [55], while more specific studies suggests that estrogen exposure decreases chemokine activity (F14), increasing the chance of developing breast cancer [56].

The present protocol also suggests that MHC class I protein binding as well as notch binding, both of which have hitherto not been studied in the context of breast cancer as more specific functional subgroups of receptor binding which are deserving of more study since both terms may provide insight into receptor binding activity during breast cancer metastasis.

Our analysis also throws up new functional clusters that should be further investigated. In C11, peptidase activity (more specifically, serine, threonine, cysteine and aspartic-type endopeptidase activity) is indicated as being associated with breast cancer. In panel C12, the focus is on protein phosphatase regulator activity (specifically protein phosphatase type 1 and 2A regulator activity) and protein phosphatase inhibitor activity. In panel C13, GTPase regulator activity instances are clubbed together, including guanyl-nucleotide exchange and Rho guanyl-nucleotide exchange factor activity. These might provide potential candidates for target discovery and therapeutics. Other functional clusters and terms included therein can be explored for relevance, as well as for detailed insight of specific molecular functions; see Additional file 5: Table S5.

### Analysis of biological processes

GO:BP analysis for Set 1 suggests that 73.5% of all proteins in this study organize themselves in 539 different process groups that participate in 205 GO:BP terms of which only 70 have hitherto been reported in the literature related to breast cancer studies (see Table 3). Details are included in Additional file 6: Table S6.

**Figure 4:**
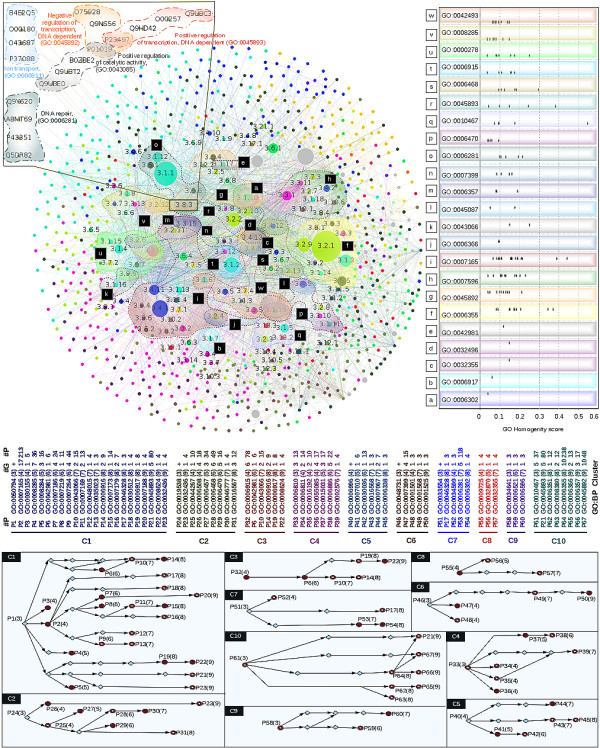
**Network of GO:BP groups of submodular network within 3rd module of West dataset.** Each submodule is highlighted by different colors and three digit number represents the BP description of each group of proteins. Process clusters are shown in the bottom, contains process id (#Id), GO terms followed by depth of the GO hierarchy in parenthesis, number of groups (#G), and number of proteins (#P). 23 GO terms common in first six module, and also those represented by gene contained in more than 2 databases, represented by dotted hull. Detail of each GO:BP term is shown in right panel. Inset represents the zoom in of a process group 3.8.3 made up of 3 overlapping and 2 independent group of proteins. Disc, ring, and rhombus represents, GO:BP terms have already been reported in literature, newly reported GO terms and cluster component not reported in our analysis (shown with symbol ‘+’), respectively.

The present protocol thus extends earlier work in suggesting novel susceptible or candidate gene targets. A representative case-study for the third module of Set 1 is shown in Figure 4. This contains the largest number of interacting proteins, namely 7712 interactions among 3187 proteins (see Table 3). Of these proteins, nearly 75% form 111 process groups that participate in 96 GO:BP terms and give 10 process clusters.

Regulation of cellular process is the largest cluster among these contains 22 predicted terms, eleven of which are known [57-67] to be involved in breast cancer and is shown in Figure 4 panel C1. These are enumerated below.

1) Signal transduction (P2).

2) Cell adhesion (P3).

3) Cell proliferation (P4).

4) Apoptosis (P6).

5) MAPK signaling pathway via the G-protein-coupled receptor (GPCR) (P7).

6) Notch signaling pathway (P8).

7) Estrogen induced cell proliferation (P5).

8) Cell death (P22).

9) Inhibition of epidermal growth factor receptor (EGFR) (P15) transcription.

10) Anti-apopotsis (P14).

11) Retinoblastoma cell apoptosis (P19).

In addition to these terms, cluster analysis also suggests eleven additional specific novel process terms that have not been explored so far in case of breast cancer. It includes small GTPase mediated signal transduction (P9), transmembrane receptor protein tyrosine kinase signaling pathway (P11), phosphatidylinositol-mediated signaling (P12), regulation of r-protein signal transduction (P13), transforming growth factor beta receptor signaling pathway (P16), regulation of JNK cascade (P17), negative regulation of transforming growth factor beta receptor signaling pathway (P18), G-protein signaling, coupled to cAMP nucleotide second messenger (P20), negative regulation of apoptotic process (P10), Positive regulation of transcription, DNA dependent (P21) and positive regulation of proteasomal ubiquitin-dependent protein catabolic process (P23). All these processes should be explored further to get additional insight into the regulation of cellular process in breast cancer.

A new candidate that has become evident through the present analysis is the Abraxas gene [68] that has two synonymous terms, FAM175A and CCDC98. Abraxas, which has recently been reported in breast cancer owing to its association with BRCA1 BRCT (BRCA1 C-terminal) repeats motif [69], links BRCA1 to a protein complex dedicated to ubiquitin chain recognition and hydrolysis at DNA double strand breaks, and is thus involved in BRCA1-dependent DNA damage response [70,71]. Abraxas and other member of this protein complex are required for the DNA damage checkpoints and cellular resistance to ionizing radiation (IR) in breast cancer [68]. Though CCDC98 is present in the GAD database, the synonymous term FAM175A is filtered out during gene network construction. The gene BRCA1 expresses two proteins P38398 and Q6IN79 in the primary network that interact with themselves as well as with four other proteins Q6UWZ7, P46736, Q96SD1 and Q9BX63 that also participate in double-strand break repair; these are in the secondary network. Q6UWZ7 is expressed by FAM175A: the two-layer protocol thus throws up a potential candidate gene that could be used as a novel target in the treatment of breast cancer. This also gives the motivation to explore other candidate genes included through the secondary network construction.The present study also finds a novel cluster, one that has not so far been studied in breast cancer and that appears to be worthy of further experimental investigation. This cluster includes the following specific biological processes: positive regulation of transcription, DNA-dependent (P21), transcription, DNA-dependent (P62), regulation of transcription, DNA-dependent (P64), transcription from RNA polymerase II promoter (P65), regulation of transcription from RNA polymerase II promoter (P66), negative regulation of transcription, DNA-dependent (P67) as a part of gene expression (P61) (Figure 4, panel C10).

### Analysis of cellular components

**Figure 5:**
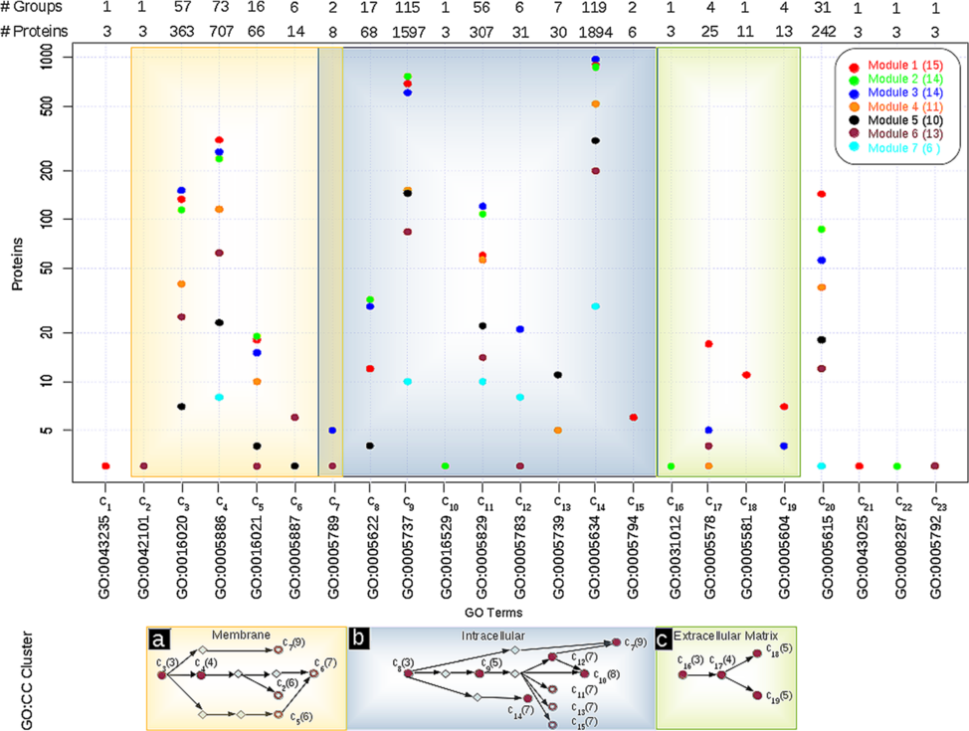
**Distribution of proteins in GO:CC terms within 7 module of West dataset.** Each dot represents the sum of proteins annotated in different class for a GO:CC term for each module, shown in different colors. Labels at the top, #Proteins, represents the cumulative sum of all the proteins for a specific GO:CC term, whereas #Group, represents the total number of group class found in all modules. Bottom panel represents the component clusters, where number in the parenthesis shows the level in the GO hierarchy. Disc, ring, and rhombus represents, GO:CC terms have already been reported in literature, newly reported GO terms and cluster component not reported in our analysis, respectively.

The distributions of proteins at various cellular locations in all seven modules of Set 1 are shown in Figure 5. About 75% of the proteins organize themselves to form 434 different component submodules that are distributed across 23 different sub-cellular locations as mentioned in Table 3; the complete details are in Additional file 7: Table S7.

The largest cluster corresponds to the intracellular component (C8) (Figure 5, panel b) which appears to be the most favorable site for breast cancer progression. The BRCA1 gene that is involved in DNA repair is mainly active in the intracellular subcomponent, in the cytoplasm (C9) of breast ductal epithelial cells. Any dysfunction in BRCA1 correlates with greater risk of breast cancer symptoms [72]. Another gene BIK, an apoptotic inducer in breast cancer cell, also known as BCL2 interacting killer performs its activity primarily at another intracellular sub-location, the endoplasmic reticulum (C12) [73]. Thapsigargin (TG), a highly specific inhibitor of the endoplasmic reticulum (C12) Ca2 + -ATPase pump and sarcoplasmic reticulum (C10), induced the apoptosis in the breast cancer cells as a subsequent to the secondary rise in [Ca2+] [74]. While the release of cytochrome-C from mitochondria (C8) served as an early signalling for the intrinsic pathway of apoptosis in breast cancer MCF-7c3 cells [75]. Caveolin-1 (CAV1), a highly conserved membrane-associated protein, is localized in the various subcellular locations including endoplasmic reticulum membrane (C7). Phosphorylation of CAV1 on Tyr-14 regulates paclitaxel-mediated apoptosis in MCF-7 breast cancer [76]. Also, various nuclear changes, such as enlargement, shrinkage, necrosis, vacuolation and pynknotic nuclei (C14) have already been reported in the breast cancer metastasis [77].

Cluster analysis also indicates more specific intracellular locations in the cytosol, mitochondrion, Golgi apparatus, sarcoplasmic reticulum and endoplasmic reticulum membrane as sites wherein breast cancer activity occurs. A recent study reports Caspase-2 releases from nucleus to cytosol, which is partially required for the apoptosis induction by taxanes in breast cancer cells [78]. Similarly, other cellular component clusters can be explored for the detailed analysis, as discussed in Additional file 7: Table S7.

Our analysis of Set 2 for all three GO categories is included in Additional file 8: Table S8, Additional file 9: Table S9 and Additional file 10: Table S10. Results for both the datasets are similar, in as much as the majority of the biological activities in BP, MF, and CC are identical in both sets. This analysis thus makes it possible to

i validate domains with relevant activity in breast cancer (as reported in literature).

ii identify new domain terms to examine details of specific biological activity, and

iii determine important domain clusters as the site of major biological activity.

## Discussion

In the past few years, the integrated analysis of gene expression data in context of PPI has received considerable attention. One objective has been to derive biologically interesting subnetworks or modules of interpretable size from large scale PPI data [79]. Subnetwork detection, in particular uncovering modules in the context of biology (similar to community detection in social networks) has received considerable attention [9,10]. In social networks such modules are shown to have a hierarchical structure with submodules being embedded in modules [80-82]. We have explored analogous organization principles in breast cancer data to construct the two-layer network of breast cancer genes and the associated proteins, using gene expression and PPI dataset.

There are, however, caveats. Noise in the available microarray or annotation data as well as incomplete annotations can make the network reconstruction ambiguous, and the complexity of the underlying regulatory mechanism, in particular the combinatorics involved makes it difficult to draw direct inferences. We have considered all currently available annotations for the given set of proteins, but since many proteins are as yet unannotated, this will affect the fraction of proteins in the largest domains and thus can alter the GO-H score for any selected set of proteins. The protocol can be modified to include a predictive aspect by using the principle of guilt-by association [83] in order to discover the possible function of a protein of unknown function: if a protein has many neighbors with a particular GO classification, it is likely to belong to that particular class as well [84,85].

## Conclusion

In summary, we have construct the two-layer “network of networks” of breast cancer genes and associated proteins. The breast cancer bilayer network has a hierarchical modular structure, with sub-modules inside modules.This structure is robust: the general topology is unaltered even when some specific proteins are removed (as seen when the LOO-CV protocol is implemented). Alternate methods that test the stability of a control network in comparison to networks formed from a random collection of genes or proteins are available, but these are not applicable here since there is extensive work on breast cancer genes and networks. Furthermore, subgroup analysis for small subgroups is unlikely to be effective [86] and randomization changes the overall topology of the control network and results in essentially no overlap with the primary network; this also reduces the probability of getting a similar prognosis among randomized groups.

The property of submodularity has advantages. Submodules are smaller units of biological organization that allow network abstraction at specific levels and thus helps determine domain activity through a small number of interacting submodular units rather than through the hundreds or thousands of proteins in a complex network. The different hub proteins within the submodules perform specific domain activities in a coordinated manner. We performed a complete ontological analysis to determine the GO profile that is involved in breast cancer. Some of these protein groups are involved in multiple biological activities and also form clusters of specific domain activities associated with various GO terms. This division into smaller groups helps to identify the set of proteins that participate in specific domain activities, and can be used to examine the organization and coordination of complex domain activities. We have also shown that specific submodules contain more conserved domain groups and these are assumed to be preserved during evolution [87].

The two-layer protocol allows not only the catalogue of the (well-studied) domain activities in breast cancer but also permits the prediction of more specific domain activities at a lower level of the ontological hierarchy. The analysis predicts new domain clusters which has not been studied in the context of breast cancer, and identifies Q6UWZ7, a protein expressed by a newly reported breast cancer marker gene Abraxas through the secondary network. In addition to identifying novel domain activities, this protocol can thus also point to new biomarkers.

Given the ever-increasing amount of interaction data available, we expect that the two-layer approach described herein will prove useful in ongoing efforts to explore the protein interaction universe and understand how domain building blocks are assembled together to perform or alter normal activity, not only in breast cancer, but in other complex diseases as well.

## Competing interests

The authors declare that they have no competing interests.

## Authors’ contributions

Conceived and design the protocol: RR, AS. Generate the results: AS, SK. Analyze the results: RR, AS, SK. Wrote the manuscript: RR, AS. Read and Approved: RR, AS, SK. All authors read and approved the final manuscript.

## Supplementary Material

Additional file 1: Table S1 Gene extracted from different data sources. Click here for file

Additional file 2: Table S2 Gene network analysis for Set 1. Click here for file

Additional file 3: Table S3 Gene network analysis for Set 2. Click here for file

Additional file 4: Table S4 Summary of topological and biological analysis of gene module and their corresponding protein module for Set 2. Click here for file

Additional file 5: Table S5 Molecular function analysis of modular network of Set 1. Click here for file

Additional file 6: Table S6 Biological process analysis of modular network of Set 1. Click here for file

Additional file 7: Table S7 Cellular component analysis of modular network of Set 1. Click here for file

Additional file 8: Table S8 Molecular function analysis of modular network of Set 2. Click here for file

Additional file 9: Table S9 Biological process analysis of modular network of Set 2. Click here for file

Additional file 10: Table S10 Cellular component analysis of modular network of Set 2. Click here for file
